# States' Performance in Reducing Uninsurance Among Black, Hispanic, and Low-Income Americans Following Implementation of the Affordable Care Act

**DOI:** 10.1089/heq.2020.0102

**Published:** 2021-07-21

**Authors:** Gregory Lines, Kira Mengistu, Megan Rose Carr LaPorte, Deborah Lee, Lynn Anderson, Daniel Novinson, Erica Dwyer, Sonja Grigg, Hugo Torres, Gaurab Basu, Danny McCormick

**Affiliations:** ^1^ Department of Medicine, Cambridge Health Alliance, Cambridge, Massachusetts, USA.; ^2^ Harvard Medical School, Boston, Massachusetts, USA.

**Keywords:** Affordable Care Act, ACA, health insurance, low-income, Hispanic, Black

## Abstract

**Purpose:** To assess state-level variation in changes in uninsurance among Black, Hispanic, and low-income Americans after implementation of the Affordable Care Act (ACA).

**Methods:** We analyzed data from the Behavioral Risk Factor Surveillance System from 2012 to 2016, excluding 2014. For Black, Hispanic, and low-income (<$35,000/year) adults 18–64 years of age, we estimated multivariable regression adjusted pre- (2012–2013) to post-ACA (2015–2016) percentage point changes in uninsurance for each U.S. state. We compared absolute and relative changes and the proportion remaining uninsured post-ACA across states. We also examined whether state-level variation in coverage gains was associated with changes in forgoing needed care due to cost.

**Results:** The range in the percentage point reduction in uninsurance varied substantially across states: 19-fold for Black (0.9–17.4), 18-fold for Hispanic (1.2–21.5), and 23-fold for low-income (1.0–27.8) adults. State-level variation in changes in uninsurance relative to baseline uninsurance rates also varied substantially. In some states, more than one quarter of Black, one half of Hispanic, and approaching one half of low-income adults remained uninsured after full implementation of the ACA. Compared with states in the lowest quintile of change in coverage, states in the highest quintile experienced greater improvements in ability to see a physician.

**Conclusions:** Performance on reducing uninsurance for Black, Hispanic, and low-income Americans under the ACA varied substantially among U.S. states with some making substantial progress and others making little. Post-ACA uninsurance rates remained high for these populations in many states.

## Introduction

Racial and ethnic minorities and low-income individuals in the United States have historically had high rates of uninsurance^1,2^ and worse access to care and health status.^1–3^ A key objective of the Affordable Care Act (ACA) was to reduce uninsurance in these medically vulnerable populations^4,5^ and thus reduce barriers to accessing care, such as physician visits. Nationwide, the ACA has resulted in a substantial decrease in uninsurance^6,7^ and improved access to and affordability^6,8^ of care among nonelderly adults, with greater gains for racial and ethnic minorities^9,10^ (57% of all ACA coverage gains),^11^ low-income individuals,^7,12,13^ and in states that expanded Medicaid.^6,7,10,12,14^ While the ACA is a federal program, states had substantial latitude in implementing many of its provisions,^15,16^ and choices made by states could have affected the state's performance in reducing uninsurance among racial and ethnic minorities and low-income populations.^17^ However, while state-to-state variation in post-ACA coverage changes have been noted for the overall population,^18,19^ little is known about individual states' performance in reducing uninsurance among racial and ethnic minorities or low-income adults under the ACA implementation.

State-to-state variation in coverage for racial/ethnic minorities and low-income individuals could have arisen from a series of state government decisions about how to implement the ACA. The ACA was designed to expand eligibility for Medicaid to all nonelderly adults earning less than 138% of the federal poverty level, a population that is disproportionately Black and Hispanic.^20^ Under the 2012 Supreme Court ruling allowing states to opt out of the ACA's Medicaid expansion, 14 states initially failed to expand their Medicaid programs.^21,22^ Additionally, after passage of the ACA, some states demonstrated a strong commitment to implementing the ACA's insurance marketplaces while other states chose to play a limited role.^17^ For example, while some states set up their own marketplaces (state exchanges), others ceded all enforcement of the ACA to federally run exchanges that have been associated with lower enrollment.^23^ States also varied substantially in the financial resources devoted to setting up their marketplaces, the robustness of efforts to educate state residents about availability of coverage under the ACA, and the number of navigators hired to assist residents in enrolling in marketplace plans.^24–26^

In this study, we used data from each U.S. state and the District of Columbia to assess the degree of variation in U.S. states' performance in reducing uninsurance for their Black, Hispanic, and low-income populations. Prior studies have established that access to care improved for each of our study populations following ACA implementation^12,27,28^; however, in this study, we also assessed whether observed state-level variation in reducing uninsurance was sufficiently large to alter the ability of Black, Hispanic, and low-income populations within states to afford a physician visit. Lastly, we assessed the proportion of each of these populations that remained uninsured in each state in the immediate post-ACA era.

## Methods

### Data source

The Behavioral Risk Factor Surveillance System (BRFSS) is a state-based telephone survey of the U.S. civilian, noninstitutionalized population conducted yearly by state health departments and the Centers for Disease Control and Prevention. The BRFSS collects data on health conditions, access to care, health insurance coverage, and demographic characteristics, including race and income for the adult population of each state and the District of Columbia. Data are collected from more than 500,000 adults annually through the use of a random-digit dialing survey methodology. Median response rates for the years studied ranged from 45% to 47%.^29^ All BRFSS data are self-reported.

### Study population

The study population consisted of two racial/ethnic cohorts, Non-Hispanic Black (*n*=108,067) and Hispanic (*n*=104,276) and one income-based cohort, any race with annual income <$35,0000 (*n*=367,284). All three cohorts were restricted to those 18–64 years of age (the target population of the ACA) residing in all U.S. states and the District of Columbia.

### Study variables

The primary outcome was the pre- to post-ACA change in uninsurance (on both the absolute and relative scales) in each U.S. state. An additional outcome was the pre- to post-ACA change in the proportion of respondents reporting foregoing a needed doctor visit in the last year due to cost (analyzed by quintile of states' change in uninsurance). For ease of interpretability, we report this outcome as the inverse, that is, not having to forgo a physician visit due to cost or, for brevity, being able to afford a doctor visit.

The primary independent variable was time, pre- versus post-ACA implementation. On January 1, 2014 the ACA's major coverage expansion provisions went into effect. We considered the years 2012–2013 to be the “pre-ACA” cohort, and 2015 and 2016 to be the “post-ACA” cohort. The 1st year of implementation, 2014, was a transitional year and thus we excluded it from our analyses (a sensitivity analysis that included 2014 had little impact on our results and is not presented further).

We obtained data on patient characteristics, including race and ethnicity, age, income, educational attainment, sex, and marital status. We classified race/ethnicity as non-Hispanic white (hereafter referred to as “white”), Hispanic, non-Hispanic Black (hereafter referred to as “Black”), or other. Age was treated as a categorical variable, beginning with ages 18–24 years, then in 5-year categories thereafter to age 64 years. Income was classified as $0–24,999, $25,000–34,999, $35,000–49,999, $50,000–74,999, and ≥$75,000. Sex and marital status were defined, respectively, as male versus female and currently married versus not currently married. We also classified each state according to its Medicaid expansion status and the year of expansion for participating states.^21^

### Statistical analyses

All analyses were conducted separately on three cohorts: Black, Hispanic, and low-income populations of each U.S. state. We chose not to focus on white–black or white–Hispanic or high-income–low-income “disparities” because our goal was to assess the actual changes in coverage experienced by these three historically medically disadvantaged populations, irrespective of the changes among whites.

We estimated the unadjusted and adjusted percentage point change in uninsurance on the absolute scale (percentage point change) from the pre- to post-ACA periods for each study cohort (Black, Hispanic, and low-income adults) in each U.S. state. We also calculated the change in uninsurance relative to each state's baseline uninsurance level. For each cohort, states were then rank-ordered according to their absolute and, separately, relative reduction in uninsurance. Adjusted estimates for percentage point changes were derived from separate multivariable logistic regression models for each study cohort that controlled for marital status, sex, employment status, and education. For analyses of the Black and Hispanic cohorts, income was also included as a covariate and for analyses of the low-income cohort, race/ethnicity was included also as a covariate in regression models. To obtain percentage point changes from logit models, we calculated predictive marginal effects at representative values by using the method of Graubard and Korn^30^ and Williams.^31^ We note that the state of Massachusetts uniquely had already implemented a comprehensive health care reform in 2006 that substantially lowered the uninsurance rate in that state before the implementation of the ACA,^32^ and thus had less opportunity for improvement under the ACA.

However, we sought to assess whether the degree of state-level variation in reductions in uninsurance were sufficiently large to have meaningful consequences for access to care across states. For this analysis, we first divided U.S. states into quintiles based on the pre- to post-ACA percentage point change in coverage. We then assessed whether quintile of state coverage change was associated with pre- to post-ACA change in ability to afford a doctor visit, using a regression modeling approach described above in a difference-in-differences framework. The difference-in-differences estimates reflect the changes in ability to afford a doctor visit in each of the top four quintiles of coverage change compared with the lowest quintile of coverage change. Lastly, using survey weights provided by the BRFSS we tabulated the number of residents of each state that remain uninsured in the post-ACA period and then calculated the proportion remaining uninsured in each state (post-ACA number uninsured/post-ACA total population) for each study cohort.

This analysis of deidentified, publicly available data is not considered human subjects research by the Cambridge Health Alliance's Institutional Review Board, and hence deemed exempt from review.

For all analyses, we used survey weights provided by the BRFSS to account for the complex sample design; all estimations were performed by using Stata, version 11 (StataCorp).

## Results

At baseline, the proportion of those in the lowest income group ($0–24,999) for Black, Hispanic, and low-income populations were, respectively, 45.2%, 51.5%, and 74.8%. Women accounted for 53.4% of the Black, 49.4% of the Hispanic, and 52.2% of the Low-Income cohorts. Other characteristics of the three study cohorts in both the pre and post-ACA periods are presented in Table 1. There were few differences between the pre and post-ACA period for all three cohorts.

**Table 1.** Characteristics of the Study Populations: 2012–2013 and 2015–2016 Behavioral Risk Factor Surveillance System, All U.S. States and Washington, District of Columbia

	Black		Hispanic		Low-income	
Characteristic	Pre-ACA, % (95% CI)	Post-ACA, % (95% CI)	Pre-ACA, % (95% CI)	Post-ACA, % (95% CI)	Pre-ACA, % (95% CI)	Post-ACA, % (95% CI)
Age, years						
18–24	17.3 (16.7 to 18.0)	17.4 (16.8 to 18.1)	19.6 (18.9 to 20.2)	19.2 (18.6 to 19.8)	19.4 (19.0 to 19.8)	18.6 (18.2 to 19.0)
25–29	10.6 (10.1 to 11.1)	10.9 (10.4 to 11.4)	12.5 (11.9 to 13.0)	12.6 (12.1 to 13.1)	12.9 (12.6 to 13.2)	12.0 (11.7 to 12.3)
30–34	11.3 (10.8 to 11.8)	11.1 (10.6 to 11.6)	14.7 (14.2 to 15.3)	14.5 (14.0 to 15.1)	11.8 (11.5 to 12.1)	12.0 (11.7 to 12.3)
35–39	9.5 (9.1 to 10.0)	10.0 (9.6 to 10.5)	11.7 (11.2 to 12.2)	11.8 (11.4 to 12.3)	8.8 (8.6 to 9.1)	9.4 (9.1 to 9.7)
40–44	11.6 (11.1 to 12.1)	11.1 (10.6 to 11.6)	12.0 (11.5 to 12.5)	11.4 (11.0 to 11.9)	9.6 (9.3 to 9.9)	9.4 (9.1 to 9.7)
45–49	10.0 (9.6 to 10.5)	9.0 (8.6 to 9.4)	8.7 (8.3 to 9.1)	8.9 (8.5 to 9.3)	8.6 (8.4 to 8.9)	8.1 (7.8 to 8.3)
50–54	12.0 (11.5 to 12.4)	11.7 (11.3 to 12.3)	8.9 (8.5 to 9.3)	9.0 (8.6 to 9.4)	10.9 (10.6 to 11.1)	10.6 (10.4 to 10.9)
55–59	9.1 (8.7 to 9.4)	9.8 (9.4 to 10.2)	6.8 (6.4 to 7.2)	6.7 (6.3 to 7.0)	9.1 (8.9 to 9.3)	9.7 (9.5 to 9.9)
60–64	8.5 (8.1 to 8.8)	8.9 (8.6 to 9.3)	5.2 (4.9 to 5.5)	5.9 (5.6 to 6.3)	8.9 (8.7 to 9.1)	10.2 (10.0 to 10.4)
Income ($)						
0–24,999	45.2 (44.4 to 46.0)	40.9 (40.1 to 41.7)	51.5 (50.7 to 52.4)	46.7 (45.9 to 47.5)	74.8 (74.5 to 75.2)	73.7 (73.3 to 74.1)
25,000–34,999	12.3 (11.7 to 12.8)	12.0 (11.5 to 12.5)	13.5 (12.9 to 14.1)	14.0 (13.4 to 14.5)	25.2 (24.8 to 25.5)	26.3 (25.9 to 26.7)
35,000–49,999	13.5 (13.0 to 14.1)	14.1 (13.5 to 14.7)	12.5 (12.0 to 13.1)	13.3 (12.7 to 13.8)	NA	NA
50,000–74,999	12.0 (11.5 to 12.5)	12.6 (12.1 to 13.2)	9.5 (9.0 to 10.0)	10.4 (9.9 to 10.9)	NA	NA
>75,000	17.0 (16.4 to 17.7)	20.4 (19.7 to 21.1)	13.0 (12.4 to 13.5)	15.7 (15.2 to 16.3)	NA	NA
Women						
	53.4 (52.6 to 54.1)	53.4 (52.6 to 54.1)	49.4 (48.6 to 50.2)	49.6 (48.9 to 50.3)	52.2 (51.8 to 52.6)	53.7 (53.2 to 54.1)
Married						
	30.0 (29.3 to 30.7)	30.3 (29.6 to 31.0)	43.7 (43.0 to 44.5)	44.5 (43.7 to 45.2)	30.3 (29.9 to 30.7)	30.5 (30.1 to 30.9)
Race/ethnicity						
White (non-Hispanic)	0		0		47.2 (46.8 to 47.6)	45.1 (44.7 to 45.6)
Black (non-Hispanic)	100		0		17.6 (17.2 to 17.9)	17.7 (17.4 to 18.1)
Hispanic	0		100		27.6 (27.1 to 28.0)	29.2 (28.7 to 29.7)
Other	0		0		7.6 (7.4 to 7.9)	7.9 (7.7 to 8.2)
Education						
Less than high school	1.7 (1.4 to 1.9)	1.4 (1.2 to 1.6)	18.8 (18.2 to 19.5)	18.6 (18.0 to 19.2)	9.0 (8.7 to 9.3)	9.4 (9.1 to 9.8)
Some high school	13.4 (12.8 to 14.0)	11.6 (11.1 to 12.2)	18.8 (18.1 to 19.5)	18.0 (17.4 to 18.6)	17.7 (17.3 to 18.1)	16.8 (16.4 to 17.2)
Completed high school	32.8 (32.1 to 33.5)	33.1 (32.3 to 33.8)	27.1 (26.4 to 27.8)	27.9 (27.3 to 28.5)	34.0 (33.6 to 34.4)	34.5 (34.1 to 35.0)
Some college	33.4 (32.7 to 34.2)	34.2 (33.4 to 34.9)	23.9 (23.2 to 24.6)	23.9 (23.3 to 24.6)	29.3 (28.9 to 29.6)	29.5 (29.1 to 30.0)
College completed	18.7 (18.2 to 19.2)	19.7 (19.2 to 20.3)	11.4 (11.0 to 11.8)	11.6 (11.3 to 12.0)	10.0 (9.8 to 10.2)	9.7 (9.5 to 9.9)
Employed						
	58.6 (57.8 to 59.3)	63.3 (62.5 to 64.0)	62.1 (61.3 to 62.9)	64.5 (63.8 to 65.2)	51.3 (50.9 to 51.7)	52.3 (51.9 to 52.8)

ACA, Affordable Care Act; CI, confidence interval; NA, not available.

### State-level variation in changes in coverage

**FIG. 1.**
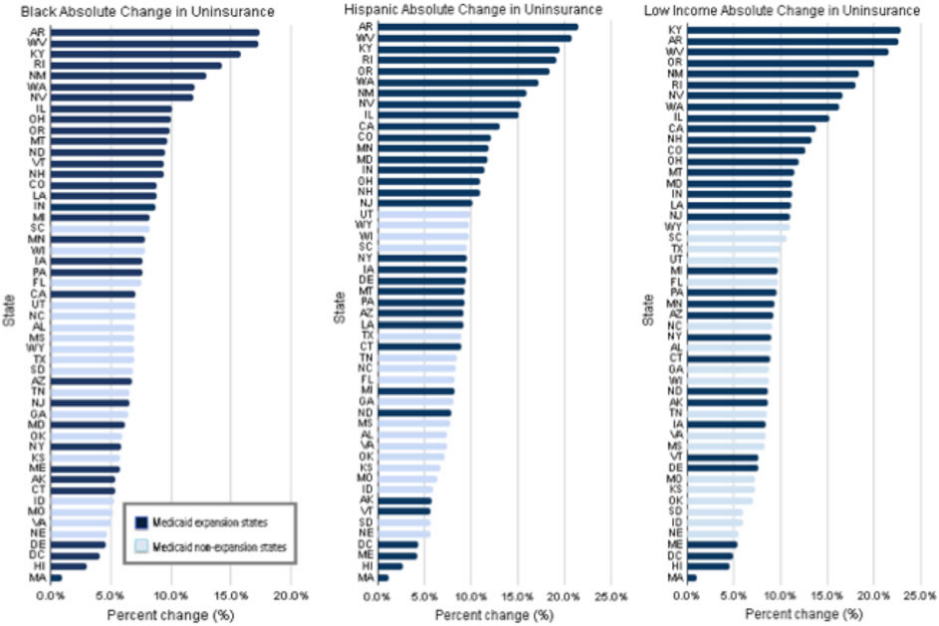
Adjusted absolute percentage point decrease in the uninsured rate among U.S. states after ACA implementation for *Black*, Hispanic, and low-income nonelderly adults: 2012–2013 to 2015–2016, BRFSS. ACA, Affordable Care Act; BRFSS, Behavioral Risk Factor Surveillance System.

**FIG. 2.**
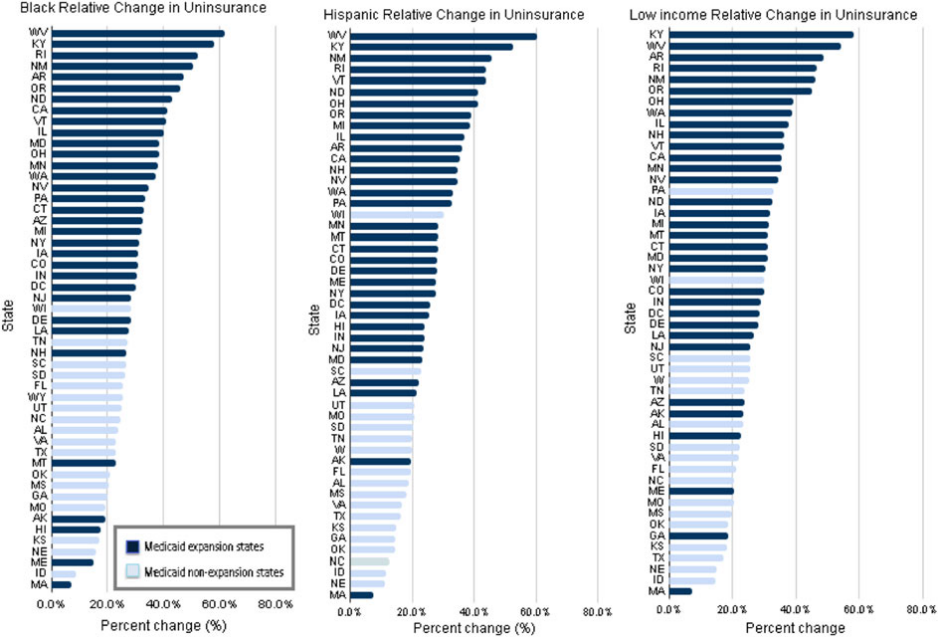
Adjusted relative decrease in the uninsured rate among U.S. states after ACA implementation for *Black*, Hispanic, and low-income nonelderly adults: 2012–2013 to 2015–2016, BRFSS.

The adjusted median reduction in uninsurance among all 50 states was 7.5 percentage points (95% confidence interval [CI], 7.1 to 7.9) for Black, 10.2 percentage points (95% CI, 9.7 to 10.7) for Hispanic, and 10.5 percentage points (95% CI, 10.0 to 11.0) for low-income adults. For each cohort, the percentage point decline in uninsurance varied substantially by state (Fig. 1). For Black individuals, the largest decline was in Arkansas, in which uninsurance dropped by 17.4 percentage points (95% CI, 14.8 to 20.1) (Fig. 1; Supplementary Appendix Table SA1). The smallest decline was in Massachusetts: 0.9 percentage points (95% CI, –0.6 to 2.5). Thus, the state with the largest percentage point decline improved 19 times more than the state with the smallest decline (similar to the state with the second smallest decline, which had not undergone a prior health reform as Massachusetts did). We also observed wide state-to-state variation in reductions in uninsurance relative to baseline (pre-ACA) uninsurance rates among Black individuals (Fig. 2), which were themselves highly variable. The median relative decline was 30.3%. The greatest relative reduction, a 61.6% decline, was achieved by West Virginia. The smallest relative reduction, 7.2% was observed in Massachusetts.

For Hispanic individuals, the largest absolute decline occurred in Arkansas where uninsurance dropped by 21.5 percentage points (95% CI, 18.5 to 24.5; Fig. 1; Supplementary Appendix Table SA2). The smallest decline was again in Massachusetts: 1.2 percentage points (95% CI, −0.8 to 3.3). Thus, absolute reductions in uninsurance across states varied by 18-fold. The median relative decline was 27.1%. The greatest relative reduction for Hispanic individuals, a 60.1% decline, was also achieved by West Virginia and the smallest relative reduction, 7.2%, was observed in Massachusetts (Fig. 2). We observed similar patterns for low-income individuals (Figs. 1 and 2; Supplementary Appendix Table SA3).

For all three study cohorts, it is visually evident (Fig. 1) that states with the largest percentage point reductions in the proportion of uninsured tended to be Medicaid expansion states. For example, among Black adults, 18 out of the 20 states that had the largest absolute reduction of uninsurance were Medicaid expansion states.

### Effect of variation in coverage across states on ability to afford a doctor visit

States achieving larger reductions in uninsurance experienced generally greater post-ACA improvements in the ability to afford a doctor visit, for each study cohort (Table 2). For Black individuals, for example, the quintile of states with the smallest change in coverage had a 4.3 percentage point improvement in the ability to afford a doctor visit (95% CI, 2.2 to 6.5), whereas the next four quintiles experienced greater improvements of 4.4 percentage points (95% CI, 2.8 to 6.1), 5.2 percentage points (95% CI, 3.5 to 6.8), 7.2 percentage points (95% CI, 5.3 to 9.1), and 12.7 percentage points (95% CI, 9.9 to 15.5). Difference-in-differences estimates were only significant for quintile 5. For Hispanic and low-income adults, we found similar patterns, but most difference-in-differences estimates were significant.

**Table 2.** Quintile of Change in Insurance Coverage and Associated Variation in Ability to See a Physician

Quintile of change	Insurance coverage rate			Able to see a physician			Difference in differences	
in coverage	Pre-ACA, % (95% CI)	Post-ACA, % (95% CI)	Change (95% CI)	Pre-ACA, % (95% CI)	Post-ACA, % (95% CI)	Change^*^ (95% CI)	estimate (95% CI)	*p*
Black								
1	76.6 (75.0 to 78.2)	81.4 (79.9 to 82.9)	4.8 (2.6 to 7.0)	76.8 (75.1 to 78.4)	81.2 (79.7 to 82.6)	4.3 (2.2 to 6.5)	Ref.	
2	78.7 (77.3 to 79.9)	86.7 (85.5 to 87.8)	8.0 (6.2 to 9.8)	79.7 (78.4 to 81.0)	84.1 (83.0 to 85.2)	4.4 (2.8 to 6.1)	0.0009 (−2.6 to 2.8)	0.94
3	72.5 (71.3 to 73.6)	82.5 (81.4 to 83.6)	10.1 (8.5 to 11.6)	75.2 (74.0 to 76.3)	80.3 (79.2 to 81.4)	5.2 (3.5 to 6.8)	0.8 (−1.9 to 3.5)	0.56
4	71.7 (70.2 to 73.2)	84.3 (83.0 to 85.5)	12.6 (10.6 to 14.5)	75.2 (73.7 to 76.6)	82.3 (81.0 to 83.5)	7.2 (5.3 to 9.1)	2.8 (−0.1 to 5.7)	0.056
5	67.6 (65.3 to 69.8)	89.5 (87.7 to 91.0)	21.9 (19.1 to 24.7)	72.3 (70.1 to 74.3)	85.0 (83.0 to 86.8)	12.7 (9.9 to 15.5)	8.4 (4.8 to 11.9)	<0.001
Hispanic								
1	72.2 (70.0 to 74.5)	71.2 (68.9 to 73.4)	−1.0 (−4.2 to 2.0)	75.8 (73.6 to 77.9)	78.1 (76.1 to 80.0)	2.3 (−0.6 to 5.3)	Ref.	
2	56.9 (55.1 to 58.7)	62.5 (60.5 to 64.4)	5.6 (2.9 to 8.2)	69.4 (67.6 to 71.1)	75.0 (73.1 to 76.7)	5.6 (3.1 to 8.1)	3.3 (−0.6 to 7.1)	0.1
3	59.6 (57.8 to 61.3)	67.7 (66.2 to 69.1)	8.1 (5.8 to 10.4)	71.6 (70.0 to 73.2)	77.7 (76.4 to 78.9)	6.0 (4.0 to 8.1)	3.7 (0.1 to 7.2)	0.04
4	50.3 (48.9 to 51.8)	61.0 (59.6 to 62.4)	10.6 (8.6 to 12.6)	69.1 (67.8 to 70.4)	75.0 (73.7 to 76.2)	5.8 (4.0 to 7.6)	3.5 (0.1 to 6.9)	0.05
5	61.3 (59.9 to 62.6)	74.7 (73.6 to 75.7)	13.4 (11.6 to 15.1)	74.0 (72.8 to 75.1)	81.1 (80.1 to 82.1)	7.1 (5.6 to 8.7)	4.8 (1.5 to 8.1)	0.004
Low income								
1	68.9 (68.1 to 69.8)	75.1 (74.2 to 75.9)	6.1 (4.9 to 7.3)	71.1 (70.3 to 71.9)	74.1 (73.3 to 75.0)	3.0 (1.8 to 4.2)	Ref.	
2	68.8 (67.6 to 70.0)	78.6 (77.7 to 79.5)	9.8 (8.4 to 11.3)	70.3 (69.1 to 71.4)	77.7 (76.8 to 78.5)	7.4 (5.9 to 8.8)	4.4 (2.5 to 6.3)	<0.001
3	54.0 (53.1 to 54.9)	65.6 (64.7 to 66.6)	11.6 (10.4 to 13.0)	63.0 (62.1 to 63.8)	68.8 (67.9 to 69.7)	5.8 (4.6 to 7.1)	2.8 (1.1 to 4.5)	0.001
4	62.5 (61.8 to 63.2)	76.9 (76.2 to 77.7)	14.4 (13.4 to 15.5)	68.2 (67.5 to 68.8)	75.7 (75.0 to 76.4)	7.6 (6.6 to 8.6)	4.6 (3.0 to 6.1)	<0.001
5	58.0 (57.0 to 59.0)	79.4 (78.5 to 80.3)	21.4 (20.1 to 22.8)	65.6 (64.6 to 66.5)	76.4 (75.5 to 77.3)	10.8 (9.5 to 12.1)	7.8 (6.0 to 9.6)	<0.001

### State variation in share of population remaining uninsured

**FIG. 3.**
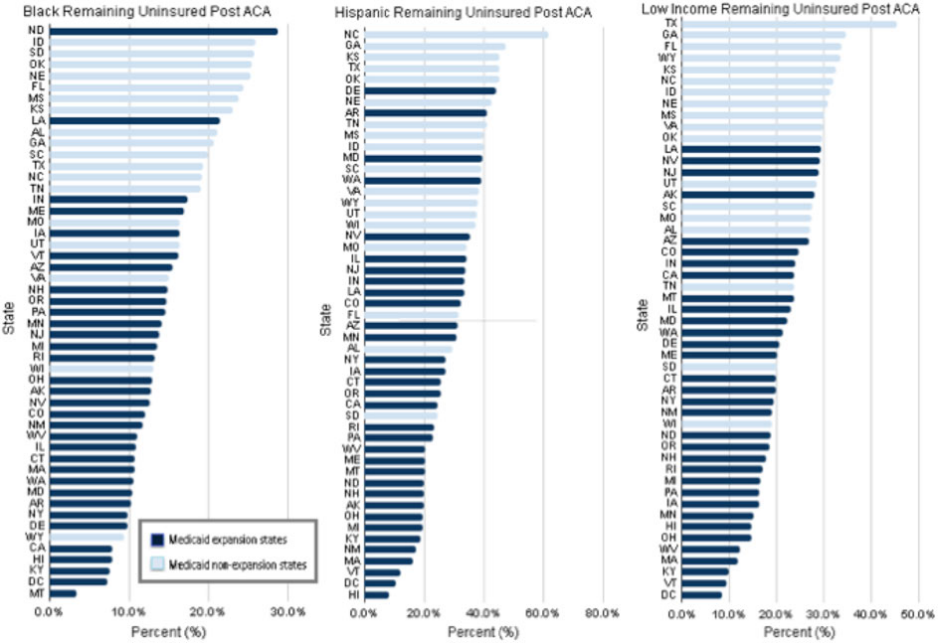
Proportion of *Black*, Hispanic, and low-income nonelderly adults remaining uninsured after ACA implementation: 2015 and 2016, BRFSS.

We found wide variation in the share of each state's population that remained uninsured in the post-ACA period for each study cohort (Fig. 3). Among Black adults, the state median share that remained uninsured was 14.5% (interquartile range, 10.7% to 19.2%); the range was 3.4% (95% CI, 0.6% to 16.2%) in Montana to 28.7% (95% CI, 19.0% to 40.8%) in North Dakota. Among Hispanic individuals, the state median share that remained uninsured was 31.6% (interquartile range, 20.4% to 39.1%); the range was 8.1% (95% CI, 5.9% to 10.9%) in Hawaii to 61.8% (95% CI, 58.1% to 65.4%) in North Carolina. Among low-income people, the state median share that remained uninsured was 23.1% (interquartile range, 18.1% to 29.1%); this rate ranged from 8.5% (95% CI, 6.1% to 11.7%) in Washington, District of Columbia to 45.3% (95% CI, 43.0% to 47.6%) in Texas.

## Discussion

Prior U.S. Census Bureau data^18,19^ and one additional analysis have demonstrated significant variation in improvements in uninsurance at the state level for the overall U.S. population following implementation of the ACA for the whole population. However, we are not aware of prior studies that have examined the ACA's differential effects on Black, Hispanic, or low-income populations across the U.S. states. In addition, we are not aware of prior studies examining the consequences of this variation on state-level variation in improvements in access to care following ACA implementation.

Our findings suggest that while the ACA resulted in improvements in coverage for Black, Hispanic, and low-income residents of every state, this benefit was strikingly uneven in its distribution across states. The performance of the best states—which cut uninsurance by ∼60%—illustrates that substantial gains in coverage for minority and low-income populations were achievable under the ACA. It also suggests that policy and implementation choices made by many of the worst performing states, which cut their uninsurance rates by only ∼15%—effectively deprived a large proportion of their minority and low-income populations from health insurance coverage that would have been available to them. In addition, our finding that states with larger improvements in reducing uninsurance had larger improvements in ability to see a physician emphasizes that the magnitude of state-level variation in coverage gains that we identified was sufficiently large to have a meaningful impact for their Black, Hispanic, and low-income populations' to access care.

In implementing the ACA, states took different approaches. Some welcomed the ACA^15,17^ and took a proactive approach to developing state-based marketplaces,^16,33^ made considerable investments in marketplace planning,^24,34^ public education and outreach efforts,^24^ hired navigators to help individuals enroll in ACA plans,^26,35^ and participated in the ACA's optional Medicaid expansion.^21,24^ Other states resisted ACA implementation, made smaller investments in these domains and rejected Medicaid expansion. We were unable to quantify the effects of states' engagement in each of these domains due to data limitations in the BRFSS; however, previous studies have demonstrated the influence of several of these factors on coverage changes overall, following the ACA.^22,23,33^ It seems likely, therefore, that the choices states made in implementing the ACA played key roles in producing the variation in performance we observed among their Black, Hispanic, and low-income populations. In particular, among the best performing states for each population, all used a state or state/federal partnership exchange (vs. a fully federally facilitated plan)^36^ and all expanded Medicaid.^21^

Another factor likely to have influenced individual states' coverage gains under the ACA is their pre-ACA coverage rates. We note that states with only small absolute improvements in coverage in our three study cohorts are of two general types: states that opposed and did not invest in policies to increase coverage and had moderate-to-high pre-ACA uninsurance rates (e.g., Nebraska, Missouri, Idaho); and those that were proactive in implementing the ACA and had low pre-ACA uninsurance rates (e.g., Massachusetts, Hawaii, District of Columbia). The latter group had much less room for improvement, likely explaining the relatively small absolute gains. For example, Massachusetts' 2006 comprehensive health reform, upon which the ACA was later modeled, had already reduced the state nonelderly adult uninsurance rate to only 4% by 2008.^32^ However, our analysis of changes in uninsurance *relative* to baseline uninsurance rates highlights substantial variation that is independent of baseline coverage rates and helps identify the particular states that performed the worst in insuring their Black, Hispanic, and low-income populations under the ACA. Factors less directly under the control of states (e.g., economic conditions and insurance market competition), may also have influenced the variation across states that we observed.

Our study has several policy implications. First, the state-level variation in coverage achieved under the ACA will likely have direct consequences for how well minority and low-income individuals are able to access and afford care during the current coronavirus disease 2019 (COVID-19) pandemic, the greatest health care crisis the United States has faced in a century. COVID-19 disproportionately affects minority^37,38^ and low income populations^38^ and COVID vaccination rates parallel these trends.^39^ The ACA largely set the coverage rates for these populations in each state before the epidemic began^7^ and will strongly influence state residents' public coverage eligibility as many workers lose employer-sponsored insurance due to COVID-19-related job losses.^40,41^

More broadly, our findings underscore the potential consequences for vulnerable populations of designing health care reforms that are largely up to individual states to implement. Political polarization among U.S. states is increasing^42,43^ and states vary substantially in the extent to which they enact social and health policies that protect the health of their vulnerable populations.^44^ The ACA was passed without significant support from congressional republicans and states with republican leadership were later substantially more likely to resist ACA implementation. Our study suggests that future health care reform options that devolve implementation authority from the federal to state governments, as the ACA has done, risk producing the same state-to-state disparities in coverage gains for minority and low-income populations as we observed in the present study.

Our study has several limitations. First, the BRFSS is a telephone survey with a response rate just under 50%, thus allowing for the possibility of nonresponse bias, although this should be mitigated by our use of BRFSS survey weights. Second, the survey does not include imprisoned or other institutionalized populations, and thus may not be representative of the entire United States. Third, BRFSS respondents self-report information on health, health insurance, and demographics, allowing for the possibility of inaccurate reporting (although this should not introduce systematic bias). However, previous studies have demonstrated that BRFSS questions have both high reliability and validity.^45–47^ Fourth, as some provisions of the ACA were implemented before the January 1, 2014 start date (e.g., extension of dependent coverage to age 26 years and because some states expanded Medicaid in previous years, our study likely underestimates actual effect sizes. Finally, as our study is observational in nature and did not include a control group, we cannot exclude the possibility that pre/post ACA changes were influenced by secular trends unrelated to the ACA, although potential changes in characteristics of residents in each state were controlled for in our multivariable analyses.

It is unclear whether the current administration's efforts to induce Medicaid non-expansion states to adopt the ACA's Medicaid expansion will be successful or whether the status quo or a more comprehensive insurance coverage expansion, such as a public option or a Medicare or All plan will prevail. However, international experience suggests that a universal, comprehensive, national health insurance program that is implemented at the national level would most effectively reduce uninsurance, particularly among minority and low-income populations in the United States, an approach now supported by some 70% of Americans.^48^ In the United States, a nationally implemented, rather than a state-implemented health insurance program could build in race-conscious, explicit mechanisms and safeguards to assure racially equitable implementation uniformly across all U.S. states.

## Supplementary Material

Supplemental data

Supplemental data

Supplemental data

## Supplementary Material

Supplementary Appendix Table SA1

Supplementary Appendix Table SA2

Supplementary Appendix Table SA3

## Abbreviations Used

ACA: Affordable Care Act
BRFSS: Behavioral Risk Factor Surveillance System
CI: confidence interval
COVID-19: coronavirus disease 2019
