# Supplementary material for: States' Performance in Reducing Uninsurance Among Black, Hispanic, and Low-Income Americans Following Implementation of the Affordable Care Act
Source: Health Equity. 2021 Jul 21;5(1):493–502. doi: 10.1089/heq.2020.0102 (PMC8317598; doi:10.1089/heq.2020.0102)
Supplement: Supplemental data [file Supp_TableS1.docx]

Appendix Table 1. Changes in Insurance Coverage After ACA implementation for Black Adults by State: 2012-2013 to 2015-2016 Behavioral Risk Factor Surveillance System, All US States and Washington DC, Ranked by Adjusted Relative Change

| **State** | **Pre-ACA Insurance Coverage Rate**  **(%)** | **Post-ACA Insurance Coverage Rate**  **(%)** | **Unadjusted Percentage Point Change in Insurance Coverage Rate** | **Adjusted Percentage Point Change in Insurance Coverage Rate** ^a^ | **Adjusted Relative Change in Uninsurance Rate^b^**  **(%)** | **Percent Remaining Uninsured Post-ACA**  **(%)** |
| --- | --- | --- | --- | --- | --- | --- |
| **WV** | 71.9 | 89.0 | 17.1 | 17.3 (14.2, 20.4) | -61.6 | 11.0 (7.4, 16.1) |
| **KY** | 72.6 | 92.4 | 19.8 | 15.8 (13.5, 18.2) | -57.9 | 7.6 (5.5, 10.5) |
| **RI** | 72.8 | 86.8 | 14.0 | 14.2 (11.1, 17.2) | -52.1 | 13.2 (9.0, 19.0) |
| **NM** | 74.3 | 88.2 | 13.9 | 13.0 (9.8, 16.3) | -50.7 | 11.8 (5.5, 23.6) |
| **AR** | 63.1 | 89.7 | 26.6 | 17.4 (14.8, 20.1) | -47.3 | 10.3 (7.1, 14.7) |
| **OR** | 78.5 | 85.3 | 6.8 | 9.9 (5.8, 14.0) | -46.1 | 14.7 (6.8, 28.8) |
| **ND** | 77.9 | 71.3 | -6.6 | 9.5 (6.1, 13.0) | -43.1 | 28.7 (19.0, 40.8) |
| **CA** | 83.2 | 92.2 | 9.0 | 7.0 (5.7, 8.2) | -41.4 | 7.8 (5.7, 10.7) |
| **VT** | 77.1 | 83.8 | 6.7 | 9.4 (5.6, 13.2) | -41.0 | 16.2 (6.1, 36.6) |
| **IL** | 74.8 | 89.2 | 14.4 | 10.1 (7.9, 12.3) | -40.0 | 10.8 (8.4, 13.8) |
| **MD** | 84.0 | 89.6 | 5.6 | 6.2 (4.6, 7.7) | -38.4 | 10.4 (8.7, 12.5) |
| **OH** | 74.0 | 87.1 | 13.2 | 10.0 (8.1, 11.8) | -38.2 | 12.9 (10.1, 16.2) |
| **MN** | 79.4 | 85.9 | 6.5 | 7.8 (6.1, 9.4) | -37.8 | 14.1 (11.5, 17.0) |
| **WA** | 67.6 | 89.5 | 21.9 | 12.0 (10.2, 13.9) | -37.1 | 10.5 (7.6, 14.3) |
| **NV** | 65.8 | 87.3 | 21.6 | 11.9 (9.0, 14.8) | -34.7 | 12.7 (8.1, 19.2) |
| **PA** | 77.1 | 85.5 | 8.3 | 7.6 (5.8, 9.5) | -33.4 | 14.5 (11.4, 18.4) |
| **CT** | 83.5 | 89.3 | 5.7 | 5.4 (3.7, 7.1) | -32.9 | 10.7 (8.5, 13.4) |
| **AZ** | 79.4 | 84.6 | 5.2 | 6.7 (4.0, 9.4) | -32.7 | 15.4 (10.3, 22.3) |
| **MI** | 74.6 | 86.5 | 11.9 | 8.2 (6.5, 9.9) | -32.1 | 13.5 (11.5, 15.8) |
| **NY** | 81.0 | 90.2 | 9.2 | 5.9 (4.4, 7.5) | -31.2 | 9.8 (8.3, 11.7) |
| **IA** | 75.5 | 83.6 | 8.1 | 7.6 (4.9, 10.2) | -30.9 | 16.4 (10.6, 24.4) |
| **CO** | 71.5 | 88.0 | 16.5 | 8.8 (7.1, 10.4) | -30.8 | 12.0 (8.6, 16.6) |
| **IN** | 71.4 | 82.6 | 11.1 | 8.7 (6.8, 10.6) | -30.5 | 17.4 (13.7, 21.8) |
| **DC** | 86.5 | 92.7 | 6.3 | 4.1 (1.4, 6.8) | -30.1 | 7.3 (5.7, 9.2) |
| **NJ** | 76.6 | 86.2 | 9.5 | 6.6 (5.0, 8.3) | -28.4 | 13.8 (11.2, 16.9) |
| **WI^c^** | 72.5 | 86.9 | 14.4 | 7.8 (5.0, 10.6) | -28.4 | 13.1 (8.3, 20.1) |
| **DE** | 83.6 | 90.2 | 6.6 | 4.6 (2.8, 6.5) | -28.2 | 9.8 (7.2, 13.1) |
| **LA** | 67.8 | 78.5 | 10.7 | 8.8 (6.3, 11.3) | -27.4 | 21.5 (18.8, 24.6) |
| **TN^c^** | 75.8 | 81.0 | 5.2 | 6.6 (4.4, 8.7) | -27.1 | 19.0 (15.5, 23.0) |
| **NH** | 64.8 | 85.2 | 20.3 | 9.4 (6.6, 12.3) | -26.8 | 14.8 (6.6, 30.0) |
| **SB^c^** | 69.2 | 80.1 | 10.9 | 8.2 (6.7, 9.8) | -26.8 | 19.9 (18.2, 21.8) |
| **SD^c^** | 73.9 | 74.3 | 0.4 | 6.8 (3.2, 10.4) | -26.2 | 25.7 (11.3, 48.6) |
| **FL^c^** | 70.7 | 75.6 | 4.9 | 7.5 (5.8, 9.3) | -25.6 | 24.4 (21.7, 27.5) |
| **WY^c^** | 72.8 | 90.6 | 17.7 | 6.9 (3.2, 10.7) | -25.5 | 9.4 (2.7, 28.2) |
| **UT^c^** | 72.0 | 83.7 | 11.6 | 7.0 (4.9, 9.1) | -25.0 | 16.3 (9.9, 25.8) |
| **NC^c^** | 71.5 | 80.8 | 9.3 | 7.0 (5.4, 8.7) | -24.7 | 19.2 (17.1, 21.6) |
| **AL^c^** | 70.9 | 78.9 | 8.0 | 6.9 (4.9, 8.9) | -23.7 | 21.1 (19.0, 23.3) |
| **VA^c^** | 77.9 | 85.0 | 7.1 | 5.1 (3.6, 6.7) | -23.1 | 15.0 (13.1, 17.2) |
| **TX^c^** | 69.7 | 80.7 | 11.0 | 6.9 (5.3, 8.5) | -22.9 | 19.3 (15.9, 23.3) |
| **MT** | 57.4 | 96.6 | 39.2 | 9.7 (5.4, 14.1) | -22.9 | 3.4 (0.6, 16.2) |
| **OK^c^** | 71.0 | 74.5 | 3.5 | 6.0 (3.9, 8.1) | -20.7 | 25.5 (20.6, 31.2) |
| **MS^c^** | 66.5 | 76.2 | 9.6 | 6.9 (4.7, 9.1) | -20.6 | 23.8 (21.6, 26.2) |
| **GA^c^** | 67.6 | 79.3 | 11.7 | 6.5 (4.4, 8.6) | -20.0 | 20.7 (18.2, 23.4) |
| **MO^c^** | 73.2 | 83.6 | 10.4 | 5.2 (3.1, 7.2) | -19.3 | 16.4 (13.4, 20.0) |
| **AK** | 72.1 | 87.2 | 15.1 | 5.4 (2.6, 8.1) | -19.2 | 12.8 (5.8, 25.8) |
| **HI** | 82.7 | 92.2 | 9.5 | 3.0 (1.1, 4.9) | -17.5 | 7.8 (2.2, 24.3) |
| **KS** | 66.4 | 76.9 | 10.5 | 5.8 (4.4, 7.2) | -17.2 | 23.1 (19.5, 27.2) |
| **NE^c^** | 70.4 | 74.7 | 4.2 | 4.7 (3.0, 6.4) | -15.7 | 25.3 (19.8, 31.8) |
| **ME** | 61.0 | 83.0 | 22.0 | 5.8 (3.3, 8.3) | -14.8 | 17.0 (8.3, 31.6) |
| **ID^c^** | 39.4 | 74.1 | 34.7 | 5.3 (2.5, 8.1) | -8.8 | 25.9 (7.1, 61.5) |
| **MA** | 87.2 | 89.3 | 2.1 | 0.9 (-0.6, 2.5) | -7.2 | 10.7 (7.7, 14.7) |

^a^Adjusted for patient characteristics including age, sex, income, marital status, educational attainment and race and ethnicity.

^b^Calculated as the percentage point reduction in uninsurance / pre-ACA uninsurance rate.

^c^Denotes Medicaid non-expansion states
