# Supplementary material for: States' Performance in Reducing Uninsurance Among Black, Hispanic, and Low-Income Americans Following Implementation of the Affordable Care Act
Source: Health Equity. 2021 Jul 21;5(1):493–502. doi: 10.1089/heq.2020.0102 (PMC8317598; doi:10.1089/heq.2020.0102)
Supplement: Supplemental data [file Supp_TableS2.docx]

Appendix Table 2. Changes in Insurance Coverage After ACA implementation for Hispanic People by State: 2012-2013 to 2015-2016 Behavioral Risk Factor Surveillance System, All US States and Washington DC, Ranked by Adjusted Relative Change.

| **State** | **Pre-ACA Insurance Coverage Rate**  **(%)** | **Post-ACA Insurance Coverage Rate**  **(%)** | **Unadjusted Percentage Point Change in Insurance Coverage Rate** | **Adjusted Percentage Point Change in Insurance Coverage Rate** ^a^ | **Adjusted Relative Change in Uninsurance Rate^b^**  **(%)** | **Percent Remaining Uninsured Post-ACA**  **(%)** |
| --- | --- | --- | --- | --- | --- | --- |
| **WV** | 65.4 | 79.5 | 14.1 | 20.8 (17.4, 24.1) | -60.1 | 20.5 (11.6, 33.6) |
| **KY** | 62.9 | 81.3 | 18.4 | 19.5 (16.3, 22.6) | -52.4 | 18.7 (10.9, 30.2) |
| **NM** | 65.1 | 82.5 | 17.4 | 15.9 (14.0, 17.8) | -45.7 | 17.5 (15.6, 19.5) |
| **RI** | 56.6 | 76.5 | 19.9 | 19.1 (15.8, 22.4) | -43.9 | 23.5 (19.5, 28.1) |
| **VT** | 87.2 | 87.9 | 0.7 | 5.6 (2.7, 8.6) | -43.9 | 12.1 (5.0, 26.3) |
| **ND** | 80.9 | 80.0 | -0.8 | 7.9 (5.1, 10.6) | -41.1 | 20.0 (12.5, 30.3) |
| **OH** | 73.1 | 80.6 | 7.5 | 11.0 (8.8, 13.3) | -41.0 | 19.4 (13.4, 27.4) |
| **OR** | 52.9 | 74.4 | 21.5 | 18.4 (15.9, 21.0) | -39.1 | 25.6 (21.3, 30.4) |
| **MI** | 78.8 | 80.6 | 1.7 | 8.2 (6.3, 10.0) | -38.6 | 19.4 (15.2, 24.6) |
| **IL** | 59.0 | 65.9 | 6.9 | 15.1 (12.2, 17.9) | -36.7 | 34.1 (30.4, 38.0) |
| **AR** | 40.2 | 59.0 | 18.8 | 21.5 (18.5, 24.5) | -36.0 | 41.0 (31.8, 50.9) |
| **CA** | 62.8 | 75.5 | 12.7 | 13.1 (11.6, 14.7) | -35.3 | 24.5 (23.3, 25.8) |
| **NH** | 68.3 | 80.1 | 11.8 | 11.0 (8.2, 13.8) | -34.7 | 19.9 (12.0, 31.1) |
| **NV** | 55.4 | 64.5 | 9.1 | 15.4 (12.1, 18.6) | -34.5 | 35.5 (31.5, 39.7) |
| **WA** | 47.8 | 61.0 | 13.2 | 17.3 (15.4, 19.2) | -33.2 | 39.0 (36.1, 42.0) |
| **PA** | 71.8 | 77.2 | 5.4 | 9.3 (7.1, 11.5) | -32.8 | 22.8 (17.4, 29.2) |
| **WI^b^** | 67.4 | 62.7 | -4.7 | 9.8 (6.5, 13.1) | -30.1 | 37.3 (30.1, 45.1) |
| **MN** | 58.2 | 69.0 | 10.7 | 11.9 (9.8, 14.0) | -28.5 | 31.0 (27.5, 34.8) |
| **MT** | 67.2 | 79.7 | 12.5 | 9.3 (7.1, 11.6) | -28.5 | 20.3 (13.5, 29.4) |
| **CT** | 68.2 | 74.4 | 6.1 | 9.0 (6.5, 11.6) | -28.5 | 25.6 (22.9, 28.6) |
| **CO** | 56.8 | 67.5 | 10.7 | 12.1 (10.3, 14.0) | -28.1 | 32.5 (30.3, 34.8) |
| **DE** | 66.3 | 56.1 | -10.1 | 9.4 (5.8, 13.0) | -27.8 | 43.9 (38.5, 49.4) |
| **ME** | 84.7 | 79.7 | -5.1 | 4.2 (2.2, 6.2) | -27.5 | 20.3 (11.7, 33.0) |
| **NY** | 65.1 | 72.9 | 7.8 | 9.6 (7.4, 11.7) | -27.4 | 27.1 (25.0, 29.3) |
| **DC** | 82.8 | 89.3 | 6.5 | 4.4 (1.3, 7.5) | -25.7 | 10.7 (6.6, 16.9) |
| **IA** | 62.4 | 73.0 | 10.6 | 9.6 (6.8, 12.3) | -25.4 | 27.0 (21.2, 33.7) |
| **HI** | 88.5 | 91.9 | 3.4 | 2.7 (1.4, 4.1) | -23.8 | 8.1 (5.9, 10.9) |
| **IN** | 51.9 | 66.4 | 14.6 | 11.5 (9.0, 13.9) | -23.8 | 33.6 (28.3, 39.2) |
| **NJ** | 57.2 | 66.2 | 9.0 | 10.1 (7.7, 12.5) | -23.5 | 33.8 (30.8, 37.0) |
| **MD** | 49.3 | 60.5 | 11.2 | 11.8 (8.8, 14.8) | -23.3 | 39.5 (34.3, 45.0) |
| **SC^c^** | 57.5 | 60.9 | 3.4 | 9.6 (7.8, 11.5) | -22.7 | 39.1 (33.2, 45.4) |
| **AZ** | 58.4 | 68.6 | 10.2 | 9.2 (6.0, 12.4) | -22.2 | 31.4 (28.5, 34.5) |
| **LA** | 57.2 | 66.5 | 9.3 | 9.2 (6.5, 11.8) | -21.4 | 33.5 (24.6, 43.7) |
| **UT^c^** | 52.1 | 62.3 | 10.2 | 9.9 (8.0, 11.8) | -20.6 | 37.7 (34.6, 40.8) |
| **MO^c^** | 68.9 | 65.7 | -3.3 | 6.4 (3.9, 8.8) | -20.5 | 34.3 (26.3, 43.4) |
| **SD^c^** | 72.4 | 75.7 | 3.3 | 5.6 (2.7, 8.5) | -20.3 | 24.3 (14.3, 38.3) |
| **TN^c^** | 57.2 | 59.0 | 1.9 | 8.5 (5.7, 11.4) | -19.9 | 41.0 (31.5, 51.1) |
| **WY^c^** | 50.6 | 62.0 | 11.4 | 9.8 (6.8, 12.8) | -19.8 | 38.0 (31.2, 45.4) |
| **AK** | 70.1 | 80.1 | 10.0 | 5.8 (3.1, 8.6) | -19.4 | 19.9 (12.0, 31.2) |
| **FL^c^** | 57.7 | 68.4 | 10.7 | 8.2 (6.3, 10.1) | -19.4 | 31.6 (29.3, 34.0) |
| **AL^c^** | 60.8 | 70.7 | 9.9 | 7.4 (5.2, 9.7) | -18.9 | 29.3 (21.1, 39.2) |
| **MS^c^** | 56.6 | 60.0 | 3.5 | 7.8 (5.3, 10.3) | -18.0 | 40.0 (27.5, 53.9) |
| **VA^c^** | 56.0 | 61.7 | 5.7 | 7.4 (5.2, 9.5) | -16.7 | 38.3 (33.9, 42.9) |
| **TX^c^** | 44.2 | 54.7 | 10.5 | 9.0 (7.0, 11.1) | -16.2 | 45.3 (43.0, 47.6) |
| **KS^c^** | 54.7 | 54.7 | 0.0 | 6.7 (5.1, 8.3) | -14.8 | 45.3 (42.4, 48.2) |
| **GA^c^** | 43.7 | 52.7 | 9.0 | 8.1 (5.5, 10.7) | -14.3 | 47.3 (41.3, 53.3) |
| **OK^c^** | 49.6 | 54.7 | 5.2 | 7.2 (4.7, 9.7) | -14.2 | 45.3 (40.0, 50.6) |
| **NB^c^** | 33.4 | 38.2 | 4.8 | 8.4 (6.4, 10.4) | -12.6 | 61.8 (58.1, 65.4) |
| **ID^c^** | 48.2 | 60.1 | 11.9 | 5.9 (2.9, 8.8) | -11.3 | 39.8 (34.4, 45.6) |
| **NE^c^** | 49.8 | 57.6 | 7.9 | 5.6 (3.5, 7.6) | -11.1 | 42.4 (38.7, 46.1) |
| **MA** | 83.0 | 83.8 | 0.8 | 1.2 (-0.8, 3.3) | -7.2 | 16.2 (13.7, 19.0) |

^a^Adjusted for patient characteristics including age, sex, income, marital status, -educational attainment and race and ethnicity.

^b^Calculated as the percentage point reduction in uninsurance / pre-ACA uninsurance rate.

^c^Denotes Medicaid non-expansion states
