# Supplementary material for: States' Performance in Reducing Uninsurance Among Black, Hispanic, and Low-Income Americans Following Implementation of the Affordable Care Act
Source: Health Equity. 2021 Jul 21;5(1):493–502. doi: 10.1089/heq.2020.0102 (PMC8317598; doi:10.1089/heq.2020.0102)
Supplement: Supplemental data [file Supp_TableS3.docx]

Appendix Table 3. Changes in Insurance Coverage After ACA implementation for Low-Income People by State: : 2012-2013 to 2015-2016 Behavioral Risk Factor Surveillance System, All US States and Washington DC, Ranked by Adjusted Relative Change.

| **State** | **Pre-ACA Insurance Coverage Rate**  **(%)** | **Post-ACA Insurance Coverage Rate**  **(%)** | **Unadjusted Percentage Point Change in Insurance Coverage Rate** | **Adjusted**^a^ **Percentage Point Change in Insurance Coverage Rate** | **Adjusted Relative Change in Uninsured Rate**  **(%)** | **Percent Remaining Uninsured Post-ACA**  **(%)** |
| --- | --- | --- | --- | --- | --- | --- |
| **KY** | 61.0 | 90.0 | 29.1 | 22.8 (20.4, 25.1) | -58.3 | 10.0 (8.4, 11.8) |
| **WV** | 60.0 | 87.7 | 27.6 | 21.6 (19.4, 23.8) | -54.0 | 12.3 (11.0, 13.8) |
| **AR** | 53.4 | 80.1 | 26.7 | 22.6 (19.5, 25.6) | -48.5 | 19.9 (17.1, 23.0) |
| **RI** | 61.3 | 83.0 | 21.7 | 18.0 (14.8, 21.2) | -46.5 | 17.0 (14.5, 19.8) |
| **NM** | 60.2 | 81.0 | 20.8 | 18.4 (16.2, 20.6) | -46.2 | 19.0 (17.1, 21.1) |
| **OR** | 55.6 | 81.6 | 26.1 | 20.0 (17.4, 22.7) | -45.0 | 18.4 (16.2, 20.7) |
| **OH** | 69.4 | 85.3 | 15.9 | 12.0 (9.9, 14.1) | -39.2 | 14.7 (13.0, 16.5) |
| **WA** | 57.9 | 78.8 | 20.9 | 16.3 (14.4, 18.1) | -38.6 | 21.2 (19.5, 22.9) |
| **IL** | 59.6 | 76.9 | 17.3 | 15.2 (12.3, 18.1) | -37.6 | 23.1 (20.8, 25.6) |
| **NH** | 63.1 | 82.2 | 19.1 | 13.3 (10.3, 16.3) | -36.1 | 17.8 (15.4, 20.6) |
| **VT** | 78.8 | 90.5 | 11.7 | 7.6 (5.5, 9.8) | -36.1 | 9.5 (8.0, 11.2) |
| **CA** | 61.0 | 76.3 | 15.3 | 13.8 (12.2, 15.5) | -35.5 | 23.7 (22.4, 25.1) |
| **MN** | 73.4 | 84.9 | 11.4 | 9.4 (7.7, 11.2) | -35.5 | 15.1 (13.9, 16.4) |
| **NV** | 51.6 | 70.9 | 19.3 | 16.6 (13.1, 20.1) | -34.2 | 29.1 (25.7, 32.7) |
| **PA** | 70.8 | 83.6 | 12.7 | 9.6 (7.4, 11.8) | -32.9 | 16.4 (14.4, 18.7) |
| **ND** | 73.2 | 81.3 | 8.1 | 8.7 (6.0, 11.4) | -31.8 | 18.7 (16.1, 21.7) |
| **IA** | 73.6 | 83.8 | 10.2 | 8.4 (6.0, 10.8) | -31.8 | 16.2 (14.0, 18.5) |
| **MI** | 69.2 | 83.4 | 14.3 | 9.7 (7.8, 11.7) | -31.6 | 16.6 (15.1, 18.2) |
| **MT** | 63.1 | 76.4 | 13.3 | 11.5 (9.0, 14.0) | -31.2 | 23.6 (21.2, 26.2) |
| **CT** | 71.4 | 80.0 | 8.6 | 8.9 (6.4, 11.4) | -31.2 | 20.0 (18.0, 22.2) |
| **MD** | 63.8 | 77.8 | 14.0 | 11.2 (8.4, 14.1) | -31.1 | 22.2 (19.3, 25.3) |
| **NY** | 70.4 | 80.7 | 10.3 | 9.0 (7.0, 11.1) | -30.5 | 19.3 (17.9, 20.8) |
| **WI^c^** | 70.7 | 81.0 | 10.4 | 8.8 (5.9, 11.7) | -30.0 | 19.0 (16.4, 21.8) |
| **CO** | 57.3 | 75.3 | 18.0 | 12.7 (10.8, 14.6) | -29.8 | 20.0 (18.0, 22.2) |
| **IN** | 61.3 | 76.1 | 14.9 | 11.2 (8.8, 13.6) | -28.9 | 23.9 (21.7, 26.1) |
| **DC** | 82.8 | 91.5 | 8.7 | 4.9 (1.6, 8.2) | -28.6 | 8.5 (6.1, 11.7) |
| **DE** | 73.1 | 79.3 | 6.3 | 7.6 (4.6, 10.6) | -28.2 | 20.7 (18.1, 23.5) |
| **LA** | 58.4 | 70.7 | 12.3 | 11.1 (8.0, 14.2) | -26.6 | 29.3 (26.5, 32.2) |
| **NJ** | 57.3 | 71.0 | 13.6 | 11.0 (8.3, 13.6) | -25.7 | 29.0 (26.4, 31.8) |
| **SC^c^** | 58.3 | 72.4 | 14.1 | 10.7 (8.6, 12.7) | -25.6 | 27.6 (25.9, 29.5) |
| **UT^c^** | 61.4 | 71.6 | 10.2 | 9.8 (8.0, 11.7) | -25.5 | 28.4 (26.3, 30.5) |
| **WY^c^** | 56.5 | 66.6 | 10.1 | 11.0 (7.6, 14.3) | -25.2 | 33.4 (29.8, 37.1) |
| **TN^c^** | 64.3 | 76.3 | 12.0 | 8.5 (5.7, 11.3) | -23.9 | 23.7 (21.3, 26.2) |
| **AZ** | 60.9 | 73.1 | 12.2 | 9.3 (6.1, 12.5) | -23.7 | 26.9 (24.4, 29.5) |
| **AK** | 63.4 | 71.9 | 8.5 | 8.6 (4.7, 12.5) | -23.5 | 28.1 (24.0, 32.5) |
| **AL^c^** | 61.5 | 73.0 | 11.5 | 9.0 (6.4, 11.5) | -23.2 | 27.0 (25.0, 29.1) |
| **HI** | 79.6 | 85.3 | 5.7 | 4.6 (2.5, 6.8) | -22.8 | 14.7 (12.9, 16.6) |
| **SD^c^** | 73.0 | 79.9 | 6.9 | 6.0 (3.0, 9.0) | -22.4 | 20.1 (17.1, 23.5) |
| **VA^c^** | 61.8 | 70.0 | 8.2 | 8.4 (5.9, 10.8) | -22.0 | 30.0 (27.8, 32.2) |
| **FL^c^** | 54.6 | 66.2 | 11.6 | 9.7 (7.5, 11.9) | -21.3 | 33.8 (32.0, 35.6) |
| **NB^c^** | 55.8 | 68.0 | 12.2 | 9.1 (7.5, 11.9) | -20.6 | 32.0 (30.1, 34.1) |
| **ME** | 73.5 | 79.9 | 6.4 | 5.4 (3.3, 7.6) | -20.5 | 20.1 (18.1, 22.3) |
| **MO^c^** | 64.3 | 72.8 | 8.5 | 7.2 (4.4, 10.0) | -20.3 | 27.2 (24.8, 29.8) |
| **MS^c^** | 58.0 | 69.8 | 11.8 | 8.3 (5.6, 10.9) | -19.7 | 30.2 (28.0, 32.5) |
| **OK^c^** | 62.7 | 70.3 | 7.6 | 7.0 (4.6, 9.4) | -18.7 | 29.7 (27.4, 32.2) |
| **GA^c^** | 52.7 | 65.4 | 12.7 | 8.8 (6.0, 11.7) | -18.7 | 34.6 (31.9, 37.4) |
| **KS^c^** | 60.4 | 67.4 | 7.0 | 7.2 (5.5, 8.9) | -18.2 | 32.6 (31.0, 34.2) |
| **TX^c^** | 42.8 | 54.7 | 11.9 | 9.9 (7.7, 12.2) | -17.3 | 45.3 (43.0, 47.6) |
| **NE^c^** | 63.5 | 69.2 | 5.7 | 5.5 (3.5, 7.5) | -15.0 | 30.8 (28.9, 32.8) |
| **ID^c^** | 59.0 | 68.7 | 9.7 | 6.0 (3.0, 9.0) | -14.7 | 31.3 (28.6, 34.0) |
| **MA** | 85.7 | 88.3 | 2.6 | 1.0 (-0.7, 2.8) | -7.3 | 11.7 (10.0, 13.6) |

^a^Adjusted for patient characteristics including age, sex, income, marital status, educational attainment and race and ethnicity.

^b^Calculated as the percentage point reduction in uninsurance / pre-ACA uninsurance rate.

^c^Denotes Medicaid non-expansion states.
